# Photobiomodulation as part of multimodal analgesia to improve pain relief and wound healing after elective caesarean section: A protocol for randomized controlled trial

**DOI:** 10.1371/journal.pone.0314010

**Published:** 2024-12-26

**Authors:** Maram Khaled, Adriana Baranov, Alvaro Diaz, Mansi Patel, Sarah Clements, Parsa Farsinejad, Kabir Khatana, Ashmitha Gnanapragasam, Sathurthika Selvanayagam, Zeineb Muhsen, Jocelyn Chan, Sanjum Hunjan, Ayman Kazi, Sapna Sharma, Lea Luketic, Joycelyne Efua Ewusie, Daniel Cordovani, Harsha Shanthanna

**Affiliations:** 1 Department of Anesthesia, McMaster University, Hamilton, Ontario, Canada; 2 Perioperative and Surgery Research Program, Population Health Research Institute, Hamilton, Ontario, Canada; 3 Michael G. DeGroote School of Medicine, McMaster University, Hamilton, Ontario, Canada; 4 Department of Obstetrics & Gynecology, McMaster University, Hamilton, Ontario, Canada; 5 Department of Health Research Methods, Evidence and Impact, McMaster University, Hamilton, Ontario, Canada; 6 The Research Institute of St. Joes Hamilton, Hamilton, Ontario, Canada; Massachusetts General Hospital, UNITED STATES OF AMERICA

## Abstract

**Background:**

Caesarean section (CS) is the most common inpatient surgical procedure performed in Canada. CS is known to cause moderate-to-severe pain, which is suggested to be associated with postpartum depression and persistent pain. Existing limitations in multimodal analgesia and conscious attempts to avoid opioids highlight the need for non-pharmacological strategies. Photobiomodulation therapy (PBMT) uses light-emitting diode (LED) and laser and has suggested potential for improving pain control and wound healing. This study aims to evaluate the effectiveness of PBMT as part of existing multimodal analgesia after elective CSs.

**Methods:**

This placebo-controlled, two-arm, multi-centre, parallel-design randomized controlled trial includes women aged ≥16 years with planned CS under spinal anesthesia (Clinical Trials Registration: NCT05738239). Patients will be randomized post-CS to intervention (n = 90) or placebo (n = 90). Study interventions will be carried out using equipment supported by Meditech International Incorporated (approved by Health Canada for pain relief). Patients will receive a maximum of 5 post-surgical treatment sessions of active PBMT (intervention: LED therapy: DUO 240 [red at 660nm and near-infrared at 840nm] applied parallel to the abdominal incision scar, followed by BIOFLEX LDR-100 laser probe (660nm red light) and the LD1-200 laser probe (825nm near-infrared light), applied at the incision wound edges) or non-effective doses of LED array and laser therapy (placebo), 4–6 hrs post-CS, and at 8am and 7pm of postoperative days 1 and 2. Patients, research assistants involved in patient recruitment and follow-up, health care providers, and data analysts will be blinded. All patients will have access to routine multimodal analgesia. Patients will be followed up in hospital on the evening of surgery and on postoperative days 1 and 2 (morning, noon, and evening); at 6 weeks; and at 3 months by telephone. Primary outcome is pain intensity with movement (elicited by asking the patient to move from supine to sitting position) using 0–10 Numerical Rating Scale (0 = no pain, 10 = worst possible pain).

**Significance:**

The results of this study may result in improved pain control, maternal satisfaction and wound healing; decrease the use of perioperative opioids; potentially decrease the incidence of postpartum depression and persistent pain; and overall lead to better postoperative outcomes thereby decreasing healthcare costs.

## Introduction

Caesarean section (CS) is the most common inpatient surgical procedure performed in Canada, constituting 31% of all births in 2021, with an average duration of hospital stay of 2.7 days [1, 2]. CS is commonly performed under neuraxial anesthesia (spinal or epidural) with the majority of elective CSs being carried out under spinal anesthesia. It is associated with moderate-to-severe pain in most women especially within the first 2 days [3, 4]. A survey of 82 pregnant women in 2005 showed that avoidance of pain during and after CS was noted to be the topmost priority [5]. Significant pain after CS not only causes maternal distress but interferes with neonatal bonding and furthermore predisposes a woman for persistent pain and postpartum depression [3].

Pain relief after CS can be inadequate [6], which may be related to insufficient use of pharmacological options, including opioids, because of concerns around neonatal safety including breast feeding. Multimodal analgesia, including acetaminophen, non-steroidal anti-inflammatory drugs and local wound infiltration, employed around the time of surgery helps to improve pain control and decrease opioid use [7]. However, these analgesics are limited by a maximum dose and may not be effective in all patients. Regional blocks such as transverse abdominal plane block or quadratus lumborum blocks are advocated; however, there is no evidence they offer additional benefit beyond neuraxial opioids [8], and can predispose women to local anesthetic toxicity [9]. Hence, there is an important need to find non-pharmacological options for pain management after CS.

Chronic postsurgical pain (CPSP), defined as pain that develops or increases after a surgical procedure and is present at 3 months or more after surgery [10], can be an important problem and can occur in up to 42% of women after CS [11]. Severe pain in the early postoperative period, preoperative depression, preoperative anxiety, and smoking can be predictive of CPSP at 3 and 6 months [12]. Although factors associated with the development of CPSP are noted to be inconsistent in studies, presence of severe pain in the first 1–2 days after surgery is a commonly identified factor [13]. CPSP can lead to significant long-term distress, suffering, continued need and potential long-term exposure to opioids, and postpartum depression (PPD). PPD is one of the most common maternal long-term complications after childbirth, with an incidence of around 13% [14]. There is altered neural response in pregnancy and postpartum due to hormonal and other influences which predisposes women to PPD and pain [15, 16]. These necessitate consideration for adjunctive therapies. History of preoperative depression and post-surgical pain is known to be associated with PPD [4, 17]. Screening for PPD is commonly performed using the 10-question Edinburgh Postnatal Depression Scale, and a score of ≥12 is considered as positive for PPD [17, 18].

The biological effects of low-level laser therapy have been studied for various clinical indications [19, 20]. The differential effects of light-emitting diodes (LEDs) in causing stimulatory effects including wound healing, epithelialization and angiogenesis, and deeper inhibitory doses of radiation by laser in modulating pain signals have been recognized [21]. The American Society for Laser Medicine and Surgery recommends the use of the term photobiomodulation (PBMT), defined as a "form of light therapy that utilizes non-ionizing forms of light sources including lasers, LEDs, and broadband light, in the visible and near infrared spectrum” [19, 22]. PBMT has been used in many musculoskeletal conditions and in some acute pain conditions [20]. Its proposed mechanism of action on pain and wound healing includes increased production of anti-inflammatory cytokines and local neo-angiogenesis. Photons emitted by PBMT are absorbed by mitochondrial chromophores leading to increased mitochondrial respiratory chain activity, which results in increased ATP levels, photodissociation of nitric oxide, increased activity of the cytochrome oxidase enzyme, and the release of reactive oxygen species and intracellular calcium. Particularly in damaged or diseased cells, with moderate levels of hypoxia, these actions result in the production of transcription factors and pro-angiogenic mediators [23, 24]. As a result, these mechanisms lead to the activation of leukocytes and macrophages, release of cytokines and stimulation of collagen synthesis, which can promote wound healing and reduce pain [25]. Despite the potential and safety, very few studies have evaluated the value of using PBMT after CS, with published studies involving small sample sizes (underpowered) or with higher potential risk of bias [26–29].

## Objectives

Our primary objective is to evaluate the effect of PBMT as part of a multimodal analgesia on postsurgical pain burden using pain scores with movement, after elective CS deliveries. Our secondary objectives are a) to evaluate the effect of PBMT on the following outcomes during the hospital stay (up to 48 hours) after surgery: 1) pain scores at rest; 2) the percentage of patients with moderate and severe pain; 3) the dose of total opioid used; 4) the incidence of opioid-related adverse effects including nausea-vomiting and sedation, and 5) patient satisfaction at hospital discharge, and b) to evaluate the effect of PBMT on the following outcomes at 6 weeks after surgery: 6) wound healing; 7) the incidence of persistent pain around the surgical site, 8) the incidence of PPD, and lastly 9) any adverse effects related to the use of PBMT at any time during the study. Our tertiary objectives include evaluation of the following outcomes at 3 months after surgery: 1) incidence of CPSP, 2) incidence of delayed wound healing or wound infection, and 3) incidence of PPD.

## Methods

This protocol is reported following the Standard Protocol Items: Recommendations for Interventional Trials (SPIRIT) 2013 and 2022 guidelines [30]. SPIRIT checklist is provided in S1 File. Study recruitment began August 18, 2023 and is expected continue until June 27, 2025.

### Design and setting

**Fig 1:**
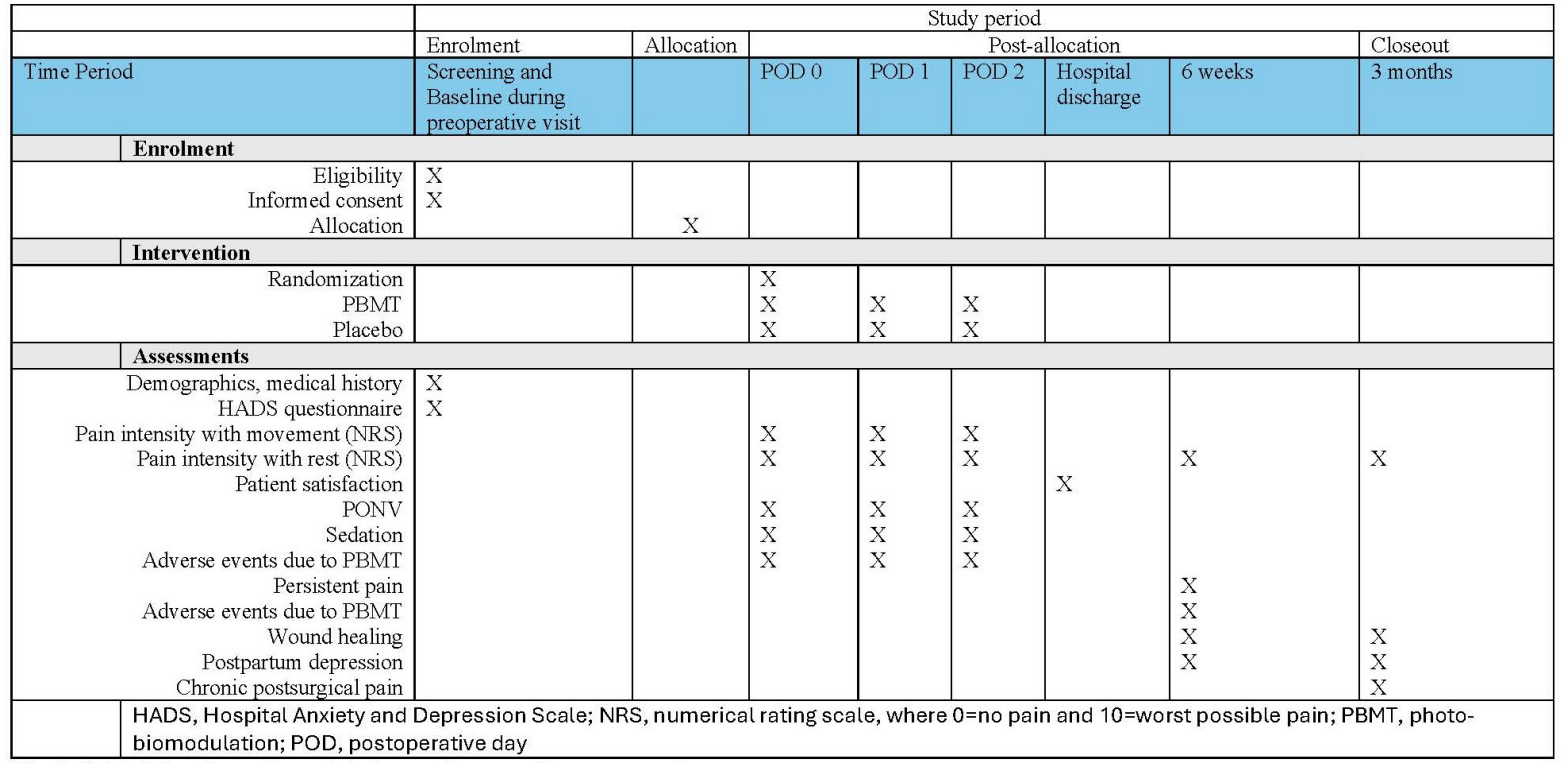
Schedule of enrolment, interventions, and assessments.

**Fig 2:**
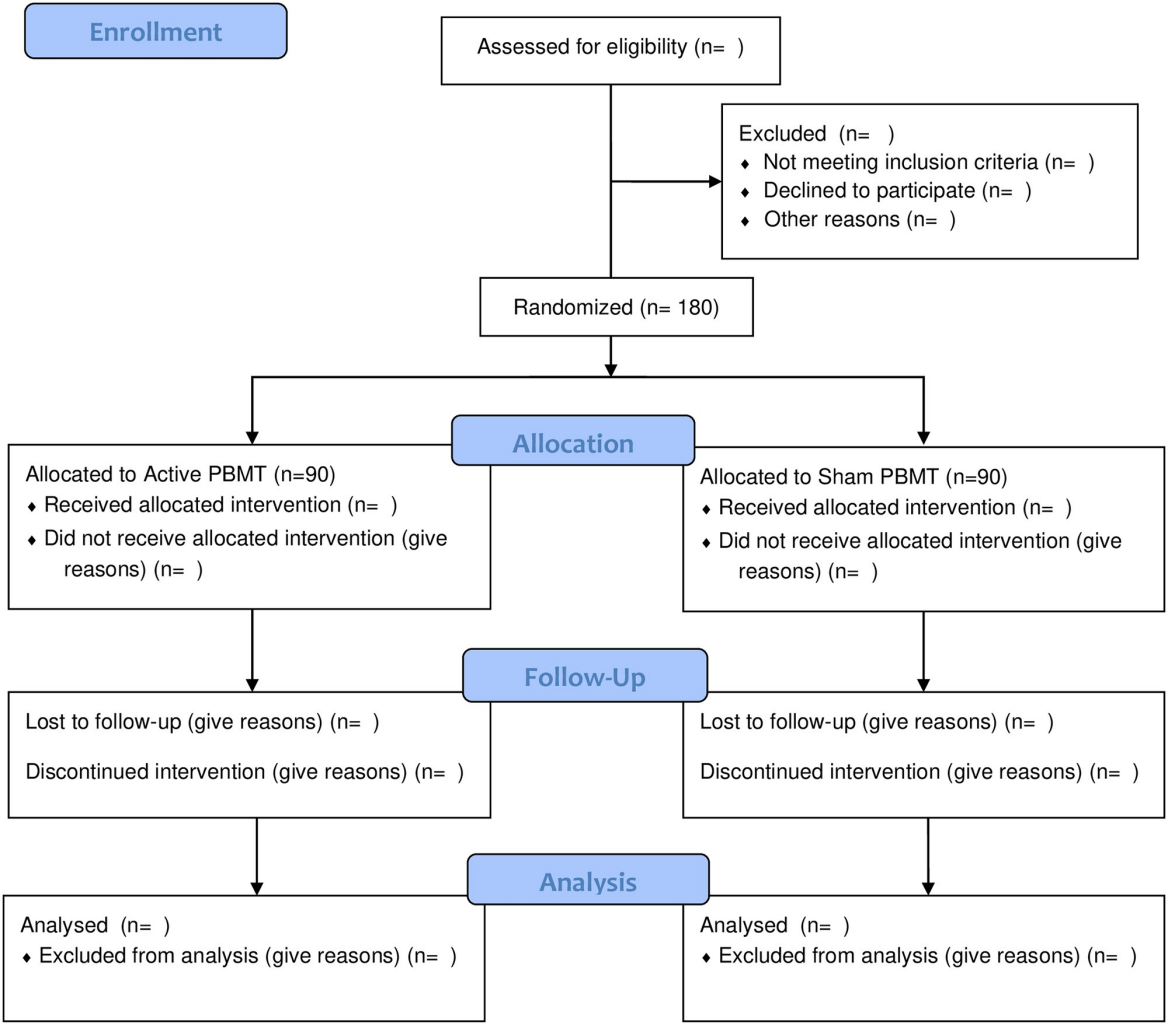
CONSORT flow diagram.

This is a placebo-controlled, multi-centre, two-arm, parallel-design, randomized controlled trial. Study workflow is shown in Fig 1, and study flow is shown in the CONSORT flow chart (Fig 2). The study will be coordinated by the Department of Anesthesia, McMaster University, and will take place at St. Joseph’s Healthcare Hamilton and McMaster University Medical Centre.

### Eligibility criteria

We will include 180 pregnant women ≥16 years old scheduled for elective CS under spinal anesthesia. Exclusion criteria are as follows: not willing, language barrier or cannot communicate in English, history of chronic ongoing pain needing regular (3 or more days per week) opioid or cannabis medications, ongoing history of substance use including alcohol, high-risk or twin/multiple pregnancy, emergency CS, CS planned under a general anesthetic or combined spinal/epidural anesthesia, and body mass index >38 kg/m^2^.

### Screening and baseline data collection

A study research assistant will approach potential participants after being introduced by the nurse caring for the patient to discuss the study, either during the preoperative meeting or early during the day of surgery. At this time, the study research assistant will obtain written informed consent and baseline study variables will be collected. Additionally, recruitment posters will be placed in the preoperative clinics and obstetric clinics.

### Randomization and allocation

Participants will be randomized soon after the C-section procedure (after confirming CS under spinal anesthesia), in a 1:1 ratio, using a computer-generated, permuted, variable block randomization, stratified by site. The personnel performing the therapy sessions (one at each site, unblinded to the study), will log on to REDCap and allocate each patient to the respective group for treatment.

### Blinding

Only personnel administering study interventions will know the study allocation, and they will not participate in any other aspects of the study. Participants, research assistants involved in patient recruitment and follow up, health care providers, nurses caring for the patient and data analysts will be blinded. The application of the intervention will not be different between the placebo and the treatment groups as the same probes will be used, with different settings. The personnel administering the intervention will set it to inactive (ineffective dose) for the placebo group, which would still light up as red. This way participants and others are blinded.

### Study interventions

The study interventions will be carried out by a trained personnel using equipment supported by Meditech International Incorporated, which has been approved by Health Canada for the use of pain control. The details of the devices and parameters being used are noted Table 1. Specifically for this study, standard operating procedures for the PBMT equipment were developed and used for training. Participants in the intervention group will have a maximum of 5 treatment sessions of PBMT; 4–6 hrs after the CS, and morning (8 am), and evening (7 pm) of postoperative day (POD) 1 and 2. Each session will involve LED therapy (7 minutes) using DUO 240 LED [red at 660 nm and near infrared at 840 nm] applied parallel to the abdominal incision scar, followed by simultaneous spot treatment using the BIOFLEX LDR-100 laser probe (660 nm red light) and the LD1-200 laser probe (825 nm near infrared light), applied at the incision wound edges, for 10 minutes. These parameters were considered as most optimal for wound healing and decreased pain based on inputs from the device manufacturer. Surgical scar and area surrounding will be considered for intervention as pain is predominantly around the incision. Participants in the control group will have 5 treatment sessions at the same timepoints, with non-effective doses of LED array and laser therapy (placebo). This way we plan to effectively blind the participants and we do not expect the non-effective dose to lead to any therapeutic effect although placebo effect cannot be ruled out. To facilitate PBMT therapy, we will be using a transparent Tegaderm dressing (3M, Canada) to cover the entire wound. PBMT equipment will be appropriately cleaned and disinfected after each therapy session.

**Table 1:** Photobiomodulation parameters to be used in the study.

	LED	LASER
Manufacturer	Bioflex Laser Therapy	Bioflex Laser Therapy
Model identifier	LED diode device (DUO+240)	Bioflex LDR-100 and LD1-200 Laser Probe
Year produced	2023	2013 and 2023
Number and type of emitters (laser or LED)	240 bicolour LED diodes	2 Laser probes Class 3b
Wavelength and band width (nm)	Red: 660nm Infra-red: 840nm	Red: 660nm Infra-red: 825nm
Pulse mode (CW or Hz, duty cycle)	Red Frequency: 50 Hz Duty Cycle 91% Infra-red CW	Red Frequency: 50 Hz Duty Cycle 91% Infra-red CW
Beam spot size at target (cm^2^)	100 cm^2^	0.1 cm^2^
Irradiance at target (mW/cm^2^)	Red: 9.1 mW/cm^2^ Infra-red: 20 mW/cm^2^	Red: 682.5 mW/cm^2^ Infra-red: 1800 mW/cm^2^
If pulsed, peak irradiance (mW/cm^2^)	Red: 9.1 mW/cm^2^	
Exposure duration (sec)	Red 180 sec Infra-red 240 sec	Red 7 sec/point total 600 sec Infra-red 7 sec/point total 600 sec
Radiant exposure (J/cm^2^)	Red: 1.64 J/cm^2^ Infra-red: 4.8 J/cm^2^	Red: 0.48 J/cm^2^ per point Infra-red: 1.26 J/cm^2^ per point
Radiant energy (J)	Red: 164 J Infra-red: 480 J	Red: 41 J Infra-red: 108 J
Number of points irradiated	Over the incision	Around the wound edges
Area irradiated (cm^2^)	100 cm^2^	0.1 cm^2^
Application technique	Contact technique. Treatment provider places the array (light side down) over the incision and leave in place for 7 minutes. The array will automatically cycle through the Red and Near Infrared wavelengths.	Treatment provider holds a laser in each hand, places a finger on the side of each nose tip to activate the beam, and directs the beam around the edges of the incision, both lasers at once, for 7 sec per point for a total of 10 minutes.
Number and frequency of treatment sessions	A maximum of 5 treatments scheduled at 1 session on the day of surgery (postop day 0), then 2 sessions on postop day 1 and 2 sessions on postop day 2.	A maximum of 5 treatments scheduled at 1 session on the day of surgery (postop day 0), then 2 sessions on postop day 1 and 2 sessions on postop day 2.
Total radiant energy over entire treatment course	644–3220 J	149–745 J

CW, continuous wave; LED, light-emitting diode

### Anesthesia management and clinical care

All participants will receive standard of care anesthesia management with spinal anesthesia along with 15 μg of fentanyl and 100 μg of morphine injected intrathecally. Based on local considerations and standard of care, participants will receive preoperative or early postoperative oral acetaminophen (975 mg), intravenous (IV) metoclopramide (10 mg), IV famotidine (20 mg) and/or 30 mL of 0.3M sodium citrate administered orally (PO). All (except participants with history of allergy to non-steroidal anti-inflammatory drugs) will have a dose of 15 mg of IV ketorolac at the end of surgery before moving into recovery. All participants will receive standard-of-care post-surgical analgesia as acetaminophen (975 mg PO Q6hrly), naproxen (500 mg Q12hrly—first dose 6 hrs after ketorolac), with either morphine (5–10 mg PO Q4hrly) or hydromorphone (2–4 mg PO Q4hrly PRN) for 48 hrs.

### Data collection and follow-up

All participants will be followed by a blinded research assistant on POD 0, POD 1, and POD 2, or until discharge, whichever comes first. Outcomes will be collected at seven time points during hospital stay; evening of surgery (8–9 pm); and on POD 1 and 2 in the morning (9–10 am), noon (12–1 pm), and evening (8–9 pm). Data collection for relevant outcomes will be conducted at 6 weeks preferably in person, to coincide with the expected follow-up visit with the physician. For patients who are unable to attend the 6-week follow-up and patients who might be under the care of midwives, we will consider a telephone follow-up. All patients will be followed at 3 months by telephone.

Study personnel collecting data from different sources will be responsible for completing the case report forms (CRFs). Clinical sites will be provided with the training needed to complete the trial CRFs prior to initiation of enrollment, as well as an instruction manual. Research personnel at each clinical site will submit the required data, as detailed on the CRFs, to the trial coordinating centre at McMaster using the REDCap electronic data capture system. Clinical site personnel will receive a unique login and password for the REDCap system and will be able to view and modify data for participants recruited at their clinical site. These CRFs will be electronic and stored within a REDCap database that will be built specifically for this study. Source documentation in relation to the trial information reported on the CRF will be filed at the Investigator’s site and made available for any trial-related monitoring, audits, REB review, and regulatory inspections when required. It is the responsibility of the study investigator to retain all study records/files in accordance with applicable regulatory requirements.

### Data safety and confidentiality

The following measures will be undertaken for data safety and confidentiality: 1) All patient information will be stored on a high security computer system and kept strictly confidential; 2) All CRFs will be identified only by a coded participant number; 3) All study participant information will be stored in locked file cabinets and accessible only to study personnel; 4) All electronic databases will be encrypted, and password protected; 5) Individual subject medical information obtained as a result of this trial is considered confidential and disclosure to third parties will be prohibited except for the following reason; 6) Medical information may be given to the subject’s personal physician or to other appropriate medical personnel responsible for the subject’s welfare; 7) Data generated as a result of the trial are to be available for inspection on request by the participating physicians, REB, and Competent Authorities; 8) If a participant revokes authorization to collect or use personal health information (PHI), the clinical site retains the ability to use all information collected before to the revocation of participant authorization. For participants who have revoked authorization to collect or use PHI, attempts will be made to obtain permission to collect at least the final clinical status (i.e., primary outcome data) at the end of their scheduled study period.

### Outcomes

The primary outcome is comparison of pain intensity with movement (from supine to sitting position) using 0–10 Numerical Rating Scale (NRS) (0 = no pain, 10 = worst possible pain) [26]. To efficiently capture pain burden over time, we will record pain scores with movement at seven time points; evening of surgery (8–9 pm); on POD 1 and 2 at morning (9–10 am), noon (12–1 pm), and evening (8–9 pm). Table 2 shows the secondary and tertiary outcomes including timepoints and measurement scales.

**Table 2:** Secondary and tertiary outcomes.

Outcome	Outcome definition and measurement	Timepoint
**Secondary outcomes**		
**Collected during hospital stay**		
Pain intensity at rest	0–10 NRS	Each of the 7 timepoints of follow-up until hospital discharge
Incidence of moderate-to-severe pain	Resting pain score of >4/10 on the NRS	Each of the 7 timepoints of follow-up until hospital discharge
Total opioid dose used in hospital	Converted to oral morphine milligram equivalents	Total dose of opioids administered at any time throughout hospital stay
Incidence of clinically important PONV	A score of ≥50 on the PONV intensity scale [31]	Any of the follow-up visits in hospital
Incidence of severe sedation	Grade 3 or above on the Pasero Opioid-induced Sedation scale [32]	Any of the follow-up visits in hospital
Patient satisfaction	0–10 scale (0 = least satisfied; 10 = most satisfied)	Hospital discharge
**Collected at 6 weeks**		
Wound healing as per the	REEDA scale (S2 File) assessed by the research assistant. REEDA refers to Redness, Edema, Ecchymosis, Discharge and Approximation. It was initially developed to assess perineal healing [33] but has been adapted to be used for abdominal wound healing following C-section [34, 35]	6 weeks follow-up
Persistent pain	Incidence elicited as Yes/No and intensity recorded using 0–10 NRS	6 weeks follow-up
Incidence of PPD. More detailed screening and appropriate clinical care.	Defined as a score of ≥12 on the EPDS [17]	6 weeks follow-up
Incidence of adverse effects	Including incidence of any infection, skin allergy, scarring, or injury or reaction to PBMT treatment	Recorded at any timepoint after surgery
**Tertiary Outcomes**		
**Collected at 3 months**		
Incidence of CPSP	As per the IASP definition [10]	3 months follow-up
Incidence of delayed or abnormal wound healing or surgical site infection	Based on patient reporting, patients will be asked about any ongoing issue and if they are being treated for it or had to see their family physician or surgeon about it. Only patients with any ongoing issue or concern will be arranged for an in-person follow up.	3 months follow-up
Incidence of PPD. More detailed screening and appropriate clinical care.	Defined as a score of ≥12 on the EPDS [17]	3 months follow-up

CPSP, Chronic postsurgical pain; EPDS, Edinburgh Postnatal Depression Scale; IASP, International Association for the Study of Pain; NRS, Numeric rating scale, where 0 = no pain and 10 = worst possible pain; PONV, postoperative nausea/vomiting; PPD, Postpartum depression

### Participant withdrawal

Participants will be provided an opportunity to withdraw at any time during the study upon their request. However, all efforts will be made to answer any questions or concerns from the participants to improve adherence to follow-up and study participation. If a participant withdraws prior to completing the trial, the research personnel will document the reason for withdrawal and attempt to collect any available outcome data. Participants will not be withdrawn from the study due to lack of adherence to the study protocol (e.g., participant received wrong intervention, missed follow-up visits). If a participant revokes authorization to collect or use personal health information (PHI), the clinical site retains the ability to use all information collected before to the revocation of participant authorization. For participants who have revoked authorization to collect or use PHI, attempts will be made to obtain permission to collect at least the final clinical status (i.e., primary outcome data) at the end of their scheduled study period. No attempts will be made to replace additional participants.

### Participants stopping their study interventions

Participants can choose to stop their study treatment(s) at any time during the course of the trial. If a participant stops their study treatment(s), they will be provided an opportunity to discuss any concerns with the local Principal Investigator (PI). If after this discussion the trial participant decides they want to resume the trial treatment (s), the Principal Investigator will re-initiate the study treatment(s) if they feel the study treatment(s) can be safely restarted. Study personnel will follow participants who decide to stop their study treatment(s) in the same way that they follow all other trial participants, unless participants opt not to be followed. The clinical investigator may negotiate a revised visit schedule in instances where the patient is unwilling to adhere to the regular schedule.

### Emergency unblinding

Based on the available literature, the study interventions do not pose a serious threat to patient perioperative care. However, in the event of an emergency situation, unblinding may be necessary or required. As the treating physician (investigator) is responsible for the medical care provided to the trial participant, the decision to break the treatment code in an emergency situation will lie solely with the site investigator. Based on the nature of requirement, the site investigator will unblind a particular patient after discussion with the research team. A telephonic access will also be provided to allow the blind to be broken as necessary. The investigator will promptly document and explain to the sponsor any premature unblinding (e.g., accidental unblinding, unblinding due to a serious adverse event) of the investigational product(s).

### Sample size considerations

Sample size was estimated based on a mixed model of repeated measures with general correlation structure [36]. A mean score of 4.7 and SD of 2 was considered for the control group [3], and a mean difference of 1 point or more in 0–10 NRS was considered as the treatment effect [4]. We believe a difference of 1 point on the NRS will be patient-important and clinically significant, based on the literature suggesting that the absolute minimal clinically important difference (MCID) for postoperative pain to be 9.9 mm on a 100 mm visual analogue scale (i.e. translating to approximately 1 point on a scale of 0–10) [37]. We expect to have 6 to 7 pain scores based on the time of patient discharge starting with 4 hrs after surgery and up to 48 hrs after surgery. Using an alpha of 0.05 and power of 90%, and an attrition of 5%, our sample size would be 90 per group. As suggested in literature, we considered low, moderate and high correlation values for repeat pain measurements and noticed no important differences. Hence, for our estimation we considered moderate correlation. Sample sizes based on varying SD, mean difference, and power are provided in Table 3. Estimations were based on the power.mmrm function in the R package long power [38]. [https://github.com/mcdonohue/longpower].

**Table 3:** Sample size estimation with moderate correlation and considering repeated measures analysis.

Number of timepoints	Mean Difference	SD per group	Power	Sample size per group	Sample size adjusted for 5% attrition
**3**	1	2	80	63	65
**3**	1	2	90	84	87
**3**	1	1.8	90	68	71
**3**	1.2	2	90	59	61
**7**	1	2	80	63	65
**7**	1	2	90	84	87
**7**	1	1.8	90	68	71
**7**	1.2	2	90	58	60

### Data analysis

Analyses will be conducted according to the intention-to-treat principle and reported as per the CONSORT guidelines. The primary outcome of pain intensity for repeated measures will be analyzed using a generalized estimating equations model, with pain scores modeled as a function of time, with the use of appropriate model and correlation structure. Multiple comparisons using Tukey contrasts will also be performed as a post hoc test. For binary outcomes, logistic regression will be undertaken to assess the effect of treatment, and a χ^2^ test will be used to calculate the p value. Continuous outcomes will be analyzed using Student’s t test for means or appropriate non-parametric tests. For all, statistical significance will be inferred, if the computed 2-sided p-value is <0.05. All analyses will be performed in R version 4.2.1.

### Ethical considerations

This protocol and informed consent form (S3 File) are approved by the Hamilton Integrated Research Ethics Board in June 2023 (project #15990). The trial will be conducted in compliance with the protocol, principles laid down in the Declaration of Helsinki, Good Clinical Practice, as defined by the International Conference on Harmonisation.

### Data and safety monitoring committee

Published studies on PBMT do not indicate any potential for the intervention to adversely affect wound healing. Considering the low risk, we will not have an independent monitor to review reportable serious adverse events (SAEs) and as such, a data and safety monitoring committee will not be used in this trial. Clinical personnel will be responsible for reporting adverse events, including SAEs via the REDCap system. This includes immediate reports followed by detailed reports and any ongoing changes. We will ensure reporting of SAEs and unanticipated problems resulting in risk to participants or others to the Research Ethics Board in accordance with reporting requirements.

### Study timeline and progress

The study was initiated in September 2023, with 66 patients enrolled to date. Considering potential recruitment of 50% of eligible participants 12–15 women/month/site for 2 sites, we expect to recruit 180 participants in 6–8 months, with final follow-ups completed by April 2025.

### Study significance

This study aims to evaluate the effectiveness of PBMT, as part of existing multimodal analgesia, for pain management and wound healing after CS, so that it can be demonstrated as appropriate for clinical use. This may result in improved maternal satisfaction and wound healing; decrease the use of perioperative opioids; potentially influence a decrease in the incidence of postpartum depression and persistent pain; and overall lead to better postoperative outcomes thereby decreasing healthcare costs.

## Supporting information

S1 File SPIRIT checklist. (PDF)

S2 File REEDA scale. (DOCX)

S3 File Study protocol and informed consent form. (PDF)
