# Supplementary material for: Photobiomodulation as part of multimodal analgesia to improve pain relief and wound healing after elective caesarean section: A protocol for randomized controlled trial
Source: PLoS One. 2024 Dec 26;19(12):e0314010. doi: 10.1371/journal.pone.0314010 (PMC11670968; doi:10.1371/journal.pone.0314010)
Supplement: S2 File — (DOCX) [file pone.0314010.s002.docx]

**REEDA SCALE FOR ASSESSMENT OF WOUND HEALING (at 6 weeks)**

| **Wound appearance** | **Scoring** | | | | **Score** |
| --- | --- | --- | --- | --- | --- |
|  | 0 | 1 | 2 | 3 |  |
| Redness | None | Mild: less than 0.5cm from each side of the wound edge | Moderate: 0.5cm to 1cm from each side of the wound edge | Severe: More than 1cm from each side of the wound edges | ______ |
| Edema | None | Mild: Less than 1cm from each side of the wound edge | Moderate: 1cm to 2cm from each side of the wound edges | Severe: More than 2cm from each side of the wound edges | ______ |
| Ecchymosis  (Discolouration of skin due to underlying blood) | None | Mild: Less than 1cm from each side of the wound edge | Moderate: 1cm to 2cm from each side of the wound edges | Severe: More than 2cm from each side of the wound edges | ______ |
| Discharge | None | Serous | Serosanguinous | Bloody and purulent | ______ |
| Approximation | Closed | Skin separation 3 mm or less | Skin and subcutaneous fat separated | Skin, subcutaneous fat and fascia separated | ______ |
| **Sum of scores** | | | | | _______ |

Alvarenga MB, Francisco AA, de Oliveira SM, et al. Episiotomy healing assessment: Redness, Oedema, Ecchymosis, Discharge, Approximation (REEDA) scale reliability. Rev Lat Am Enfermagem 2015;23(1):162-8. doi: 10.1590/0104-1169.3633.2538 [published Online First: 2015/03/26]
