# Supplementary material for: Photobiomodulation as part of multimodal analgesia to improve pain relief and wound healing after elective caesarean section: A protocol for randomized controlled trial
Source: PLoS One. 2024 Dec 26;19(12):e0314010. doi: 10.1371/journal.pone.0314010 (PMC11670968; doi:10.1371/journal.pone.0314010)
Supplement: S3 File — (PDF) [file pone.0314010.s003.pdf]

Title: Application of photo-biomodulation as part of multimodal analgesia to improve pain relief and wound healing for patients having elective C section: a randomized controlled trial

Principal Investigator
Dr Harsha Shanthanna

Protocol Number: PBMT V2.0 / 2023-04-10
Clinical Trials Registration: NCT05738239
Study Sponsor: Department of Anesthesia, McMaster University and Research Institute of St Joseph's Hospital

Project Coordination
Department of Anesthesia, McMaster University

To ensure transparency and scientific integrity, this protocol is being submitted as a supporting document along with our study protocol manuscript for publication in the journal PLOS One

Funding:

- Southern Ontario Pharmaceutical and Health Innovation Ecosystem (SOPHIE) program, supported through the Government of Canada via the Federal Economic Development Agency for Southern Ontario (FedDev)
- Hamilton Academic Health Sciences Organization

STUDY SUMMARY

Title: Application of photo-biomodulation as part of multimodal analgesia to improve pain relief and wound healing for patients having elective Caesarean section: a randomized controlled trial

Primary Objective: To evaluate the effect of photo-biomodulation therapy (PBMT) as part of a multimodal analgesia on post-surgical pain burden using pain scores with movement, after elective Caesarean section (CS).

Secondary Objectives: 1) To evaluate the effect of PBMT on the following post-surgical outcomes recorded during the hospital stay (up to 48 hours): pain scores at rest; percentage of patients with moderate and severe pain; dose of total opioid used in mg of oral morphine equivalents; and incidence of opioid-related adverse events including nausea-vomiting, and sedation. 2) To evaluate the effect of PBMT on the following outcomes at 6 weeks after surgery: wound healing; incidence of patients with persistent pain; incidence of post-partum depression (PPD) and incidence of any adverse effects related to the use of PBMT.

Tertiary Objective: To evaluate the effect of PBMT on the following outcomes at 3 months after surgery: the incidence and intensity of chronic post-surgical pain, incidence of delayed wound healing or wound infection at 3 months after surgery; and the incidence of PPD.

Design: Placebo-controlled two-arm parallel-design randomized controlled trial

Inclusion Criteria: Women aged ≥16 years with planned CS under spinal anesthesia

Exclusion Criteria: One or more of the following: not willing; language barrier or cannot communicate in English; history of chronic ongoing pain needing regular (3 or more days per week) opioid or cannabis medications, ongoing history of substance use including alcohol, high risk or twin/multiple pregnancy, emergency CS, CS planned under a general anesthetic or combined spinal epidural anesthesia.

Coordination & Sites: Department of Anesthesia, McMaster University; St Joseph's Healthcare Hamilton, and McMaster University Medical Centre.

Sample Size: 180 patients

Randomization: Patients will be randomized on the day of surgery and soon after the C-section procedure (after confirming CS under spinal anesthesia), in a 1:1 ratio, using a computer-generated, permuted, variable block randomization, stratified by site.

Allocation: Research nurse performing the therapy sessions (one at each site unblinded to the study), will log on to REDCap and allocate each patient to the respective group for treatment.

Blinding: Patients, research assistants involved in patient recruitment and follow up, health care providers, nurses caring for the patient and data analysts will be blinded.

Study interventions

Standardized Anesthesia and Pain Management: All patients will have spinal anesthesia and multimodal analgesia, which will include local wound infiltration, oral acetaminophen, NSAIDs, and as-needed opioids.

Study Interventions: Carried out using equipment supported by Meditech International Incorporated, which has been approved by Health Canada.

Study Group: Patients will have 5 treatment sessions of PBMT provided by a trained research nurse; 4 hrs after the CS, then morning (8 am), and evening (7 pm) of postoperative day (POD) 1 and 2. Each session will involve LED therapy (7 minutes) using DUO 240 LED (red at 660 nm and near infrared at 840 nm) applied parallel to the abdominal incision scar, followed by simultaneous spot treatment using the BIOFLEX LDR-100 laser probe (660 nm red light) and the LD1-200 laser probe (825 nm near infrared light), applied at the incision wound edges, for a total treatment time of 10 minutes.

Placebo Group: Patients will have 5 treatment sessions at the same time, with non-effective doses of LED array and LASER therapy.

Follow-up: All patients will be followed for 7 time points during their hospital stay either until discharge or up to 48 hours after surgery; at 6 weeks coinciding with their expected follow-up visit with their physician; and at 3 months by a phone call.

LAY ABSTRACT

C-section is the most common surgical procedure performed in Canada. Despite using a combination of medications (Tylenol, anti-inflammatories and rescue opioids) routinely, it can cause significant pain causing maternal distress that can interfere with newborn bonding. Untreated pain is known to contribute to postpartum depression and persistent pain. Considering the safety of the newborn and side effects, opioids are preferentially avoided. In view of all these reasons, there is a greater need for non-pharmacological approaches for pain relief. Photo-biomodulation therapy (PBMT) includes Low level LASER (works on pain) and light-emitting diodes (LED) (works on wound healing). In this study 180 pregnant women planned for elective C-section will be randomized to receive active PBMT and the other half to receive inactive PBMT. All patients will receive standard anesthesia management and pain medications. PBMT will be administered by a trained research nurse in 5 sessions (2 times/day) up to discharge. Pain scores will be collected 3 times a day for 48 hours or up to discharge, along with other outcomes including total opioid used, maternal satisfaction, wound healing, and persistent pain, postpartum depression, and wound healing at 6 weeks and 3 months.

1. BACKGROUND and RATIONALE

1.1 Burden of acute pain after Caesarean section

Caesarean section (CS) is the most common inpatient surgical procedure performed in Canada, with an average duration of stay of 2.7 days.(1) Its rate continues to increase; from 28.2% in 2016–2017 to 31.0% in 2020–2021.(1, 2) CS is commonly performed under neuraxial anesthesia (spinal or epidural) with majority of elective CSs being carried out under spinal anesthesia. It is associated with moderate to severe pain in most women and is more severe within the first 2 days. In a large cohort study involving 1288 parturients including 391 women having caesarean delivery, the mean pain score (standard deviation) was 4.7 (2.0).(3) In another prospective study of 195 women having elective CS under spinal anesthesia, the median (interquartile range) visual analogue score (0–100 scale) with movement was 53 (32–72).(4) Avoidance of pain during and after CS was noted to be the topmost priority among women.(5) Significant pain after CS not only causes maternal distress but interferes with neonatal bonding and furthermore predisposes a woman for persistent pain and postpartum depression.(3)

1.2 Multimodal analgesia for Caesarean section

It is possible that inadequate pain relief after CS is related to insufficient use of pharmacological options including opioids because of concerns around neonatal safety including breast feeding. A 2013 Cochrane review on oral analgesia after CS identified only 13 low-quality studies with all reporting inadequate pain relief.(6) To be more effective and to minimize opioids, multimodal analgesia is commonly employed around the time of surgery.(7) Considering the higher risk of opioid-related adverse effects such as nausea-vomiting, itching and sedation, an intrathecal dose of 50-100 µg morphine is preferred, injected along with spinal local anesthetic. Other analgesics commonly used as part of the multimodal analgesia include acetaminophen (Tylenol), non-steroidal anti-inflammatory drugs and local wound infiltration. All of these are limited by a maximum dose to be used and may not be effective in all patients. Although multiple regional blocks such as transverse abdominal plane block or quadratus lumborum blocks are advocated, meta-analyses of studies have indicated no additional benefit beyond obtained by the neuraxial opioid,(8) and can predispose women to local anesthetic toxicity.(9) Hence, there is an important need to look for non-pharmacological options.

1.3. Chronic post-surgical pain and Postpartum depression

Chronic or persistent pain after CS can be an important problem. As per the International Association for the Study of Pain (IASP), chronic post-surgical pain (CPSP) is defined as pain that develops on increases after a surgical procedure as identified at three months or more after surgery; localized to the area of surgery or projected to the innervation territory; and pain that is not explained by an infection, malignancy, a pre-existing pain condition or any other alternative cause (10). A prospective study of 527 women noted an incidence of 18.3 %, 11.3 % and 6.8 %, respectively at 3, 6 and 12 months, and observed that more severe pain during movement within 24 h of surgery and preoperative depression were predictive of pain persistence at 6 months (11). A more recent prospective study of 462 women (among 621) notes an incidence of 25.5% (95% CI: 21.8–29.7) at 90 days. Presurgical anxiety (adjusted relative risk [RR] 1.03; 95%CI: 1.01–1.05), smoking (adjusted RR 2.22; 95%CI: 1.27–3.88) and severe pain in the early postoperative

period (adjusted RR 2.79; 95%CI: 1.29–6.00) were predictive of CPSP (12). Overall, the incidence of CPSP after CS can vary between 4% to 41.8% and generally decreases over time (13). Although factors associated with its development are noted to be inconsistent in studies, presence of severe pain in the first 1-2 days after surgery can be noted as the most commonly identified factor (13). CPSP can lead to significant maternal distress, suffering, continued need and potential long-term exposure to opioids, and post-partum depression (PPD). PPD is one of the commonest maternal long-term complications after childbirth, with an incidence of around 13%, as noted in published studies (14) and also in a large observational study (15). History of preoperative depression and post-surgical pain can influence the incidence of PPD (3, 15). In a longitudinal study of 1288 women, there was a threefold increase in the odds of PPD (at 8 weeks) in women having severe acute postpartum pain (3). In studies, screening for PPD is commonly performed using the Edinburgh Postnatal Depression Scale consisting of 10 questions, and a threshold of ≥12 is considered as positive for PPD (15, 16).

1.4 Photo-biomodulation therapy (PBMT) and pain relief

Biological effects of low-level LASER therapy have been studied for various clinical indications.(17,18) Over the years the differential effects of light emitting diodes (LEDs) in causing stimulatory effects including wound healing, epithelialization and angiogenesis, and deeper inhibitory doses of radiation by LASER in modulating pain signals have been recognized.(19) As per the American Society for Laser Medicine and Surgery the term photo-biomodulation should be used, and PBMT is defined as a "form of light therapy that utilizes non-ionizing forms of light sources including LASERS, LEDs, and broadband light, in the visible and near infrared spectrum".(17, 20) PBMT has been used in many musculoskeletal conditions and in some acute pain conditions.(18) Its proposed mechanism of action on pain and wound healing includes increased production of anti-inflammatory cytokines and local neo-angiogenesis apart from other actions (Figure 1).

Figure 1: Mechanisms of photobiomodulation therapy

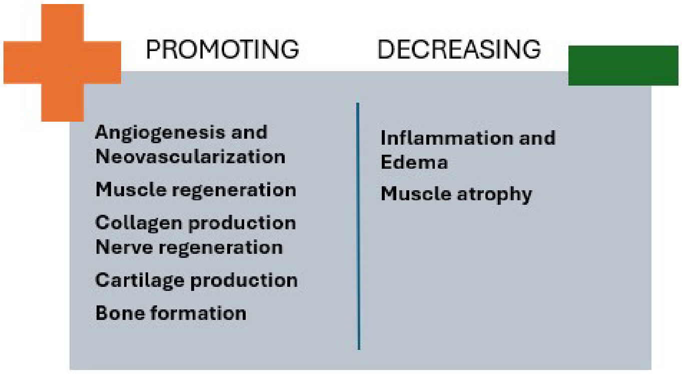

1.5 Literature review of non-pharmacological modalities for Caesarean section

We identified two systematic reviews evaluating the role of non-pharmacological modalities on after CS.(21, 22) Dutra et al looked at comparative studies involving both vaginal and CS delivery and observed only two studies using PBMT after vaginal delivery.(21) In a more recent (2020) Cochrane review, Zimpel et al looked at complementary therapies for post-caesarean pain.(22) Because of low sample sizes, potential risk of bias and heterogeneity among included trials, they were unable to conclude any findings with confidence for all interventions considered including music, acupressure, and others. They were unable to find trials using LASER therapy specifically.(23) Independent of these reviews, we identified two randomized controlled trials (RCTs) using PBMT for CSs.(24, 25) In a recent 4-arm RCT, Araujo et al compared 2 different doses of PBMT along with a control and placebo PBMT in 88 patients and reported that pain scores were reduced in the PBMT arm at 44–48 hrs after surgery. But the trial was underpowered for multiple arm testing with only 22 patients in each arm.(24) In another small RCT (n=20), Mokmeli et al observed that PBMT after CS has no deleterious effects on lactation.(25) Despite known potential and safety, there have not been any well-designed and large RCTs to assess the benefits of PBMT on postsurgical pain after CS.

2. OBJECTIVES

2.1 Primary objective

To evaluate the effect of PBMT as part of a multimodal analgesia on post-surgical pain burden using pain scores with movement, after elective caesarean deliveries.

2.2 Secondary objectives

To evaluate the effect of PBMT on the following outcomes during the hospital stay (up to 48 hours) after surgery: 1) Pain scores at rest; 2) The percentage of patients with moderate and severe pain; 3) The dose of total opioid used in mg of oral morphine equivalents; 4) The incidence of opioid-related adverse including nausea-vomiting and sedation, and 5) Patient satisfaction at hospital discharge.

To evaluate the effect of PBMT on the following outcomes at 6 weeks after surgery: 6) wound healing; 7) The incidence of persistent pain around the surgical site, 8) The incidence of PPD, and lastly 9) Any adverse effects related to the use of PBMT at any time during the study.

2.3. Tertiary objectives

In all patients, to evaluate the following outcomes at 3 months after surgery: 1) incidence of chronic post-surgical pain, 2) incidence of delayed wound healing or wound infection at 3 months after surgery, and 3) incidence of PPD.

3. METHODS

3.1 Design

Placebo-controlled two-arm parallel-design randomized controlled trial. Study flow is indicated in the CONSORT flow chart (Figure 2)

3.2. Inclusion criteria

Pregnant women ≥16 years scheduled for elective CS under spinal anesthesia

3.3. Exclusion criteria

One or more of the following: not willing, language barrier or cannot communicate in English, history of chronic ongoing pain needing regular (3 or more days per week) opioid or cannabis medications, ongoing history of substance use including alcohol, high risk or twin/multiple pregnancy, emergency CS, CS planned under a general anesthetic or combined spinal epidural anesthesia.

3.4 Screening and Baseline data collection

At each site there are typically 2-3 women booked for elective CS each day, with approximately 8-10 surgeries per week. Patients attend to their preoperative anesthesia meeting a few days in advance, but this would involve a busy day for these patients and may not be conducive to support a separate research meeting for consenting in a comfortable and private area. All patients will arrive a few hours before their elective C-surgery at the labour and delivery OR suite. A study research assistant will approach patients after being introduced by the nurse caring for the patient to discuss about the study. Patients willing to participate will be consented along with collection of baseline study variables.

Figure 2: Consort Flow Chart

CONSORT 2010 Flow Diagram

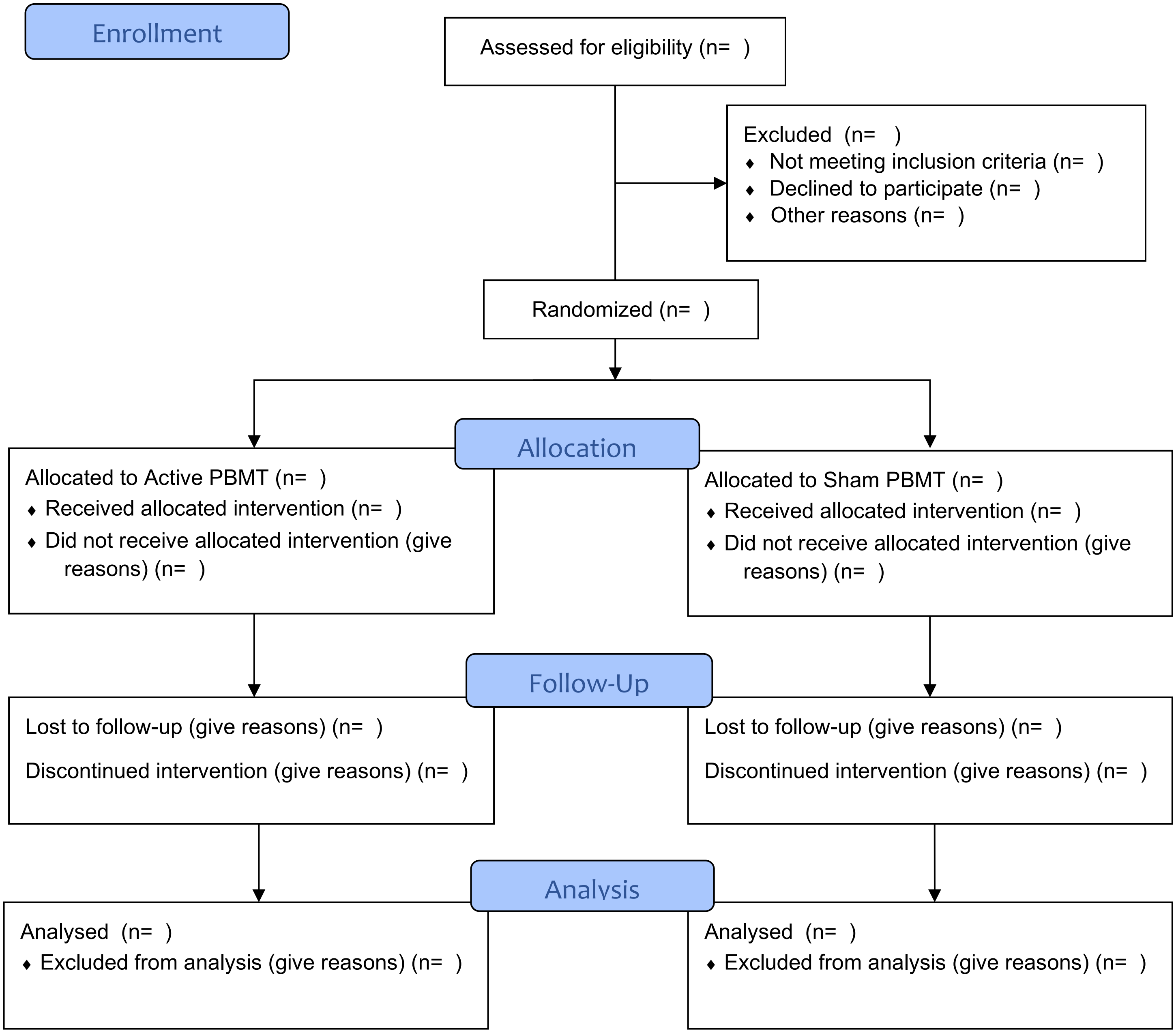

3.5 Coordination & Sites

The study will be coordinated by the Department of Anesthesia, McMaster University, and take place at St. Joseph's Hospital (SJH) and McMaster University Medical Centre (MUMC).

3.6 Randomization and Allocation

Patients will be randomized on the day of surgery and soon after the C-section procedure (after confirming CS under spinal anesthesia), in a 1:1 ratio, using a computer-generated, permuted, variable block randomization, stratified by site. Research nurse performing the therapy sessions (one at each site unblinded to the study), will log on to REDCap and allocate each patient to the respective group for treatment.

3.7 Blinding

Patients, research assistants involved in patient recruitment and follow up, health care providers, nurses caring for the patient and data analysts will be blinded.

3.8 Study Interventions

The study interventions will be carried out by a trained research nurse using equipment supported by Meditech International Incorporated, which has been approved by Health Canada. At each site, the nurse will perform these sessions as per the standard operating procedures developed specifically for this study (Appendix 1).

3.8.1 Study Group

Patients will have 5 treatment sessions of PBMT provided by a trained research nurse; 4-6 hrs after the CS, then morning (8 am), and evening (7 pm) of postoperative day (POD) 1 and 2. Each session will involve LED therapy (7 minutes) using DUO 240 LED [red at 660 nm and near infrared at 840 nm] applied parallel to the abdominal incision scar, followed by simultaneous spot treatment using the BIOFLEX LDR-100 laser probe (660 nm red light) and the LD1-200 laser probe (825 nm near infrared light), applied at the incision wound edges, for a total treatment time of 10 minutes.

3.8.2 Placebo Group

Patients will have 5 treatment sessions at the same time, with non-effective doses of LED array and LASER therapy.

3.9 Data Collection and Follow-Up

All patients will be followed by a blinded research assistant for 7 time points during their hospital stay and relevant outcomes will be collected; evening of surgery (8-9 pm); on POD 1 and 2 at morning (9-10 am), noon (12-1 pm), and evening (8-9 pm). All patients will also be contacted at 6 weeks in person, to coincide with their expected follow-up visit with their physician and relevant outcomes will be collected. At 3 months, all patients will be followed up by a phone call, and an in-person visit if required.

3.10 Participant Withdrawal

No patients will be withdrawn during the study, unless requested by the patient. Participants are allowed to withdraw consent for trial participation at any time during the trial. If a participant withdraws prior to completing the trial, the research personnel will document the reason for withdrawal and attempt to collect any available outcome data. Participants will not be withdrawn from the study due to lack of adherence to the study protocol (e.g., participant received wrong intervention, missed follow-up visits). If a participant revokes authorization to collect or use personal health information (PHI), the clinical site retains the ability to use all information collected before to the revocation of participant authorization. For participants who have revoked authorization to collect or use PHI, attempts will be made to obtain permission to collect at least the final clinical status (i.e., primary outcome data) at the end of their scheduled study period. No attempts will be made to replace additional participants.

3.11 Patients Stopping their Laser Treatment

Patients can choose to stop their study treatment(s) at any time during the course of the trial. If a patient stops their study treatment(s), they will be provided an opportunity to discuss any concerns with the local PI. If after this discussion the trial participant decides they want to resume the trial treatment (s), the Principal Investigator will re-initiate the study treatment(s) if they feel the study treatment(s) can be safely restarted. Study personnel will follow patients decide to stop their study treatment(s) in the same way that they follow all other trial participants, unless patient opts not to be followed. The clinical investigator may negotiate a revised visit schedule in instances where the patient is unwilling to adhere to the regular schedule.

3.12 Anesthesia Management and Clinical Care

Standard of care anesthesia management: All women will have their surgery under spinal anesthesia along with 15 µg of fentanyl and 100 µg of morphine injected intrathecally. All will have a preoperative dose of acetaminophen 975 mg along with 30 mL of 0.3 M sodium citrate orally administered 30 minutes before the procedure, along with 10 mg of IV metoclopramide administered using a 50 or 100 ml normal saline bag. All (except patients with history of allergy to NSAIDs or ketorolac) will have a dose of 15 mg of ketorolac IV at the end of surgery before moving into recovery. Standard-of-care post-surgical analgesia: All patients will continue to have 975 mg PO acetaminophen Q6hrly along with naproxen 500 mg Q12hrly (first dose 6 hrs after ketorolac), with either morphine 5-10 mg PO Q4hrly or hydromorphone 2-4 mg PO Q4hrly PRN for 48 hrs.

3.13 Emergency Unblinding

Based on the available literature, the study interventions do not pose a serious threat to patient perioperative care. However, in the event of an emergency situation, unblinding may be necessary or required. As the treating physician (investigator) is responsible for the medical care provided to the trial participant, the decision to break the treatment code in an emergency situation will lie solely with the site investigator. Based on the nature of requirement, the site investigator will unblind a particular patient after discussion with the research team. A telephonic access will also be provided to allow the blind to be broken as necessary. The investigator will

promptly document and explain to the sponsor any premature unblinding (e.g., accidental unblinding, unblinding due to a serious adverse event) of the investigational product(s).

4. ASSESSMENT OF OUTCOMES

4.1 Primary Outcome

Comparison of pain intensity with movement (elicited by asking the patient to move from supine to sitting position) using 0-10 Numerical Rating Scale (NRS) (0=no pain, 10=worst possible pain). To efficiently capture pain burden over time, we will record pain scores with rest and movement at seven time points; evening of surgery (8-9 pm); on POD 1 and 2 at morning (9-10 am), noon (12-1 pm), and evening (8-9 pm). A 0 to 10 NRS is suggested by the Initiative on Methods, Measurement, and Pain Assessment in Clinical Trials (IMMPACT) (26).

4.2 Secondary Outcomes

Outcomes recorded during their hospital stay:

1. Pain intensity at rest. Similar to pain recording with movement, we will record resting pain scores at seven time points after surgery and up to discharge using a 0-10 NRS

2. Percentage of patients with moderate to severe pain (non-responders). Patients with average resting pain score of >4/10 during their hospital stay.

3. Total opioid dose used in hospital: All opioids used will be converted into oral morphine milligram equivalents for comparison.

4a. Incidence of patients with clinically important postoperative nausea/vomiting (PONV), measured using PONV intensity scale on POD 0, and POD 1 and 2 morning visits. To be positive we will consider if patient has clinically important PONV in any of the visits. PONV intensity scale has been validated to provide an intensity scale and also distinguish clinically important PONV (27)

4b. Incidence of severe sedation. Patients with grade 3 and above in the Pasero Opioid-induced Sedation scale (grade 3= frequently drowsy, arousable but drifts off to sleep during conversation) (28), observed during any of the follow up visits in hospital.

5. Patient satisfaction at hospital discharge measured using a 0-10 scale (0=least satisfied; 10=most satisfied).

Outcomes recorded at 6 weeks after surgery:

6. Wound healing as per the REEDA scale (Appendix 2) assessed by the research assistant. REEDA refers to Redness, Edema, Ecchymosis, Discharge and Approximation. It was initially developed to assess perineal healing (29) but has been adapted to be used for abdominal wound healing following C-section (30, 31).

7. Incidence of persistent pain, elicited as Yes/No and intensity recorded using 0-10 NRS.

8. Incidence of PPD, considered as positive if the Edinburgh Postnatal Depression Scale (EPDS) score is ≥12 at 6 weeks (15). For any study participant, if the EDPS indicates the possibility of

PPD, we will inform the respective obstetrical team and suggest for more detailed screening and appropriate clinical care.

9. Incidence of adverse effects due to PBMT recorded at any time point after surgery, including incidence of any infection, skin allergy, scarring, or injury or reaction to PBMT treatment.

4.3. Tertiary outcomes

Outcomes recorded at 3 months after surgery by a phone call or in person meeting as appropriate.

1. Incidence of CPSP, this will be as per the IASP definition (10)

2. Incidence of delayed or abnormal wound healing or surgical site infection based on patient reporting (we expect most follow up visits to be a virtual visit) as Yes or No. Patients will be asked about any ongoing issue and if they are being treated for it or had to see their family physician or surgeon about it. Only patients with any ongoing issue or concern will be arranged for an in-person follow up.

3. Incidence of PPD, considered as positive if the EPDS score is ≥12 at 3 months. For any study participant, if the EDPS indicates the possibility of PPD, we will inform the respective obstetrical team and suggest for more detailed screening and appropriate clinical care.

Summary table of outcomes and measurements

Primary Outcome	**Measurements**	**Analysis plan**
Pain intensity with movement during the hospital stay or first 48 hrs after surgery, using 0-10 Numerical Rating Scale (NRS)	At seven time points; evening of surgery (8-9 pm); on POD 1 and 2 at morning (9-10 am), noon (12-1 pm), and evening (8-9 pm).	Compared using generalized estimating equations model (GEE), with pain scores modeled as a function of time, with the use of appropriate model and correlation structure
Secondary Outcomes		
Pain intensity at rest during the hospital stay or first 48 hrs after surgery, using 0-10 Numerical Rating Scale (NRS)	At seven time points; evening of surgery (8-9 pm); on POD 1 and 2 at morning (9-10 am), noon (12-1 pm), and evening (8-9 pm).	Compared using generalized estimating equations model (GEE), with pain scores modeled as a function of time, with the use of appropriate model and correlation structure
Incidence of moderate to severe pain during the hospital stay or first 48 hrs after surgery (non-responders).	Patients with average resting pain score of >4/10 during their hospital stay	Adjusted logistic regression model
Total opioid dose used in hospital during the hospital stay or first 48 hrs after surgery	All opioids used will be converted into oral morphine milligram equivalents	Adjusted linear regression model

Incidence of clinically important PONV during the hospital stay or first 48 hrs after surgery	Clinically important PONV as described using the PONV intensity scale	Adjusted logistic regression model
Incidence of severe sedation during the hospital stay or first 48 hrs after surgery	Patients with grade 3 and above in the Pasero Opioid-induced Sedation scale (grade 3= frequently drowsy, arousable but drifts off to sleep during conversation)	Adjusted logistic regression model
Patient satisfaction at hospital discharge	Measured using a 0-10 scale (0=least satisfied; 10=most satisfied).	Adjusted logistic regression model
Wound healing	Measured using REEDA scale: the total score ranges from 0 to 15, with lower scores representing better wound healing.	Adjusted linear regression model
Incidence of with persistent pain at 6 weeks	Elicited as Yes/No and intensity recorded using 0-10 NRS.	Adjusted logistic regression model
Incidence of PPD at 6 weeks	Using EPDS with a threshold of ≥12	Adjusted logistic regression
Incidence of adverse effects due to PBMT recorded at any time point after randomization and hospital discharge	Such as infection, scarring, skin allergy or injury or reaction to PBMT treatment.	Adjusted logistic regression model
Tertiary Outcomes		
Incidence of CPSP at 3 months after surgery	As per the IASP definition	Adjusted logistic regression model
Incidence of PPD at 3 months after surgery	Using EPDS with a threshold of ≥12	Adjusted logistic regression model
Incidence of delayed or abnormal wound healing at 3 months	Patients will be asked about any ongoing issue and evaluated with a physician follow up if appropriate.	Adjusted logistic regression model

5. STUDY TIMELINE

We expect the study to be initiated around the end of April 2023.

Planning phase: 3-4 months Meeting of investigators; Finalization of protocol; Finalizing study contracts; Development of study aids; Approval by ethics committee; Randomization scheme; Site initiation and Training; Study registration; and Publication of protocol

Recruitment phase: 6-8 months Considering potential recruitment of 50% of eligible participants 12-15 women/month/site for 2 sites, 180 participants in 6-8 months

Final follow-up: 1.5 months All patients with full 6 weeks follow up

Completion phase: 2-3 months Data analysis and Publication of results

All phases: 17-18 months

6. STUDY SIGNIFICANCE

With C-section being the most common inpatient surgical procedure performed in Canada, interventions to improve its outcomes have larger and universal impact. C-section is known to cause moderate to severe pain and women rank avoidance of pain during and after caesarean delivery as their highest priority. Furthermore, large cohort studies have observed an association between acute postoperative pain and postpartum depression as well as persistent pain. Considering the existing limitations in multimodal analgesia and a concerted need to avoid opioids in this context, there is a greater need for non-pharmacological strategies. Photo-biomodulation therapy (PBMT) can improve pain control and wound healing. By using PBMT after surgery, we have the potential to achieve better pain control and postoperative outcomes. The study aims to evaluate the effectiveness of PBMT as part of existing multimodal analgesia, so that it can be demonstrated as appropriate for clinical use. This may result in improved maternal satisfaction and wound healing; decrease the use of perioperative opioids; potentially influence a decrease in the incidence of postpartum depression and persistent pain; and overall lead to better postoperative outcomes thereby decreasing healthcare costs.

7. SAMPLE SIZE CONSIDERATIONS

Sample size was estimated based on a mixed model of repeated measures with general correlation structure (32). A mean score of 4.7 and SD of 2 was considered for the control group (3) and a mean difference of 1 point or more in 0-10 NRS was considered as the treatment effect. Using an alpha of 0.05 and power of 90%, and an attrition of 5%, our sample size would be 90 per group.

8. DATA ANALYSIS

According to the intention-to-treat principle and reported as per the CONSORT guidelines. Primary outcome of pain intensity for repeated measures will be analyzed using a generalized estimating equations model (GEE), with pain scores modeled as a function of time, with the use of appropriate model and correlation structure. Binary outcomes will be analyzed using a $\chi 2$ or Fisher's test and continuous outcomes will be analyzed using Student's t test for means or appropriate non-parametric test. For all, statistical significance will be considered using a 2-sided test with p-value is <0.05. All analyses will be performed in R version 4.2.1

9. ETHICAL CONSIDERATIONS

This trial will be conducted in compliance with the protocol, principles laid down in the Declaration of Helsinki, Good Clinical Practice (GCP), as defined by the International Conference on Harmonisation (ICH). This protocol will be reviewed and approved by the Hamilton Integrated Research Ethics Board (HiREB) prior to commencement of the trial.

10. DATA MANAGEMENT

Study personnel collecting data from different sources will be responsible for completing the case report forms (CRFs). Clinical sites will be provided with the training needed to complete the

trial CRFs prior to initiation of enrollment, as well as an instruction manual. Research personnel at each clinical site will submit the required data, as detailed on the CRFs, to the trial coordinating centre at McMaster using the REDCap electronic data capture system. Clinical site personnel will receive a unique login and password for the REDCap system and will be able to view and modify data for participants recruited at their clinical site. These CRFs will be electronic and stored within a REDCap database that will be built specifically for this study. Source documentation in relation to the trial information reported on the CRF will be filed at the Investigator's site and made available for any trial-related monitoring, audits, REB review, and regulatory inspections when required. It is the responsibility of the study investigator to retain all study records/files in accordance with applicable regulatory requirements.

10.1 Data Quality and Integrity

The Data Management Plan will outline the procedures to ensure data quality and will include the following:

1. All research personnel will undergo a training session prior to trial commencement to ensure consistency in trial procedures including data collection and reporting;
2. The Project Office personnel will review detailed monthly reports on screening, enrollment, patient follow-up, data transmission, thoroughness, and completeness of data collection, and event rates, and they will rapidly address any identified issues;
3. Any change or correction to a CRF should be dated, initialed, and explained (if necessary) and should not obscure the original entry (i.e., an audit trail should be maintained).
4. The REDCap system can be built to use a variety of mechanisms for checking data at the time of entry including skip logic, range checks, and data type checks.
5. Such periodic audits to identify any errors or omissions within the collected data will be performed and notify the sender and data management assistants of any such issues;
6. The Research Coordinator will communicate to investigators about regular quality control reports and audits.

10.2 Confidentiality and Unblinded Data

The following measures will be undertaken for data safety and confidentiality.

1. All patient information will be stored on a high security computer system and kept strictly confidential.
2. All CRFs will be identified only by a coded participant number
3. All study participant information will be stored in locked file cabinets and accessible only to study personnel.
4. All electronic databases will be encrypted, and password protected.
5. Individual subject medical information obtained as a result of this trial is considered confidential and disclosure to third parties will be prohibited except for the following reason.
6. Medical information may be given to the subject's personal physician or to other appropriate medical personnel responsible for the subject's welfare.
7. Data generated as a result of the trial are to be available for inspection on request by the participating physicians, REB, and Competent Authorities.
8. If a participant revokes authorization to collect or use personal health information (PHI), the clinical site retains the ability to use all information collected before to the revocation

of participant authorization. For participants who have revoked authorization to collect or use PHI, attempts will be made to obtain permission to collect at least the final clinical status (i.e., primary outcome data) at the end of their scheduled study period.

10.3 Data and Safety Monitoring Committee

Data safety monitoring committees (DSMCs) are generally recommended for any controlled trial that will compare rates of mortality or morbidity. Guidance from the Federal Drug Administration supports that a DSMC is not needed for clinical trials exploring interventions to promote symptom relief (http://www/fda.gov/RegulatoryInformation/Guidances/ucm12 069.htm), which is our primary outcome. Published studies on PBMT does not indicate any potential for the intervention to adversely affect wound healing. Considering the low risk, we will have an independent monitor to review REB reportable SAEs and as such, a DSMC will not be used in this trial

10.4 Data and Record Maintenance

As per section 76 of the Health Canada Natural Health Products Regulations, the sponsor shall maintain complete and accurate records to establish that the trial is conducted in accordance with GCP guidelines and regulations. The qualified investigator will immediately, on discontinuance of the clinical trial by the sponsor, in its entirety or at a clinical trial site, notify both the clinical trial subjects and the research ethics board of the discontinuance, and provide them with the reasons for the discontinuance and advise them in writing of any potential risks to the health of clinical trial subjects or other persons. The sponsor shall maintain all records referred to the conduct of this trial for a period of 25 years.

11. SAFETY AND REPORTING

11.1 Definitions

11.1.1 Adverse Events

As per the ICH guidelines, an adverse event is any untoward medical occurrence that may present during treatment, but which does not necessarily have a causal relationship with the treatment.

11.1.2 Serious Adverse Event

A serious adverse event (SAE) is any adverse event leading to any of the following:

- Fatal
- Life threatening
- Requires or prolongs hospital stay
- Results in persistent or significant disability or incapacity
- A congenital anomaly or birth defect
- An important medical event

11.1.3 Suspected unexpected serious adverse reactions

Suspected unexpected serious adverse reactions (SUSARs) are events that meet the following criteria:

• Suspected to be causally associated with any study medication
• Unexpected if the nature, severity, or outcome of the reaction(s) is not consistent with the reference information (i.e., Investigator's Brochure)
• Serious (as defined above for an SAE)
• Not an efficacy outcome as defined in outcome section
Note: Study outcomes will be recorded separately and not be reported as SAEs, except if, because of the course or severity or any other feature of such events, the investigator, according to his/her best medical judgment, considers these events as exceptional in this medical condition. Hospitalizations, which were planned before inclusion in the study (e.g., elective or scheduled surgery or other interventions arranged prior to the start of the study), will not be regarded as SAEs. This pertains also to hospitalizations that are ambulant (<12 hours) or are part of the normal treatment or monitoring of the studied disease or another disease present before inclusion in the study and which are not due to a worsening of the disease.

11.1.4 Clinical Site Reporting

Clinical personnel at each site will be responsible for reporting adverse events, including SAEs, to the coordinating centre via the REDCap system. Immediate reports followed by detailed reports and any ongoing changes will be communicated to the coordinating centre via the REDCap Cloud system. The coordinating centre will ensure reporting of SAEs and unanticipated problems resulting in risk to participants or others to their local REB/IRB by the sponsors, in accordance with local reporting requirements. Copies of each report and documentation of ethic board notification and receipt will be kept in the clinical site's study file. The sponsor shall submit to the regulatory authorities all safety updates and periodic reports, as required by applicable regulatory requirement(s).

12. PARTNER SUPPORT

This investigator-initiated study is supported in-kind by Meditech International Incorporated, who are providing the PBMT equipment needed for the study. There is no expectation of any regulatory approvals based on the study results for Meditech International Incorporated.

13. TRIAL COORDINATION AND SPONSOR

Trial will be sponsored by the Department of Anesthesia, McMaster University and Research Institute of St Joes, Hamilton. It will be coordinated by the Department of Anesthesia – Research Office, McMaster University, 1280 Main Street West, MDCL 2109, Hamilton, ON L8S 4K1; Tel: 905-525-9140 ext. 21737, Fax: 905-523-1224

14. SCHEDULE OF VISITS

Time Period	Screening and Baseline during preoperative visit	Post-surgery POD 0	POD 1	POD 2	Hospital discharge	In-person follow up at 6 weeks after surgery	Telephone or in-person follow up at 3 months
Eligibility	X						
Informed consent	X						
Demographics	X						
Medical history							
HADS questionnaire	X						
Active medications	X						
Randomization		X					
PBMT treatment / sham		X	X	X			
Pain intensity with movement (NRS)		X	X	X			
Pain intensity with rest (NRS)		X	X	X		X	X
Patient satisfaction					X		
PONV		X	X	X			
Sedation		X	X	X			
Adverse events due to PBMT		X	X	X			

Persistent pain						X	
Adverse events due to PBMT						X	
Wound healing						X	X
Postpartum depression						X	X
Chronic postsurgical pain							X

15. REFERENCES

1. Information CIoH 2022;Pages. Accessed at CIHI at https://www.cihi.ca/en/hospital-stays-in-canada#:~:text=In%202020%E2%80%932021%2C%20the%20rate,28.2%25%20in%202016%E2%80%932017. on January 19 2023.
2. Gu J, Karmakar-Hore S, Hogan ME, Azzam HM, Barrett JFR, Brown A, et al. Examining Cesarean Section Rates in Canada Using the Modified Robson Classification. J Obstet Gynaecol Can. 2020;42(6):757-65.
3. Eisenach JC, Pan PH, Smiley R, Lavand'homme P, Landau R, Houle TT. Severity of acute pain after childbirth, but not type of delivery, predicts persistent pain and postpartum depression. Pain. 2008;140(1):87-94.
4. Guevara J, Carvalho JCA, Downey K, Ye XY, Sharkey AM, Arzola C. Predicting pain after Cesarean delivery: pressure algometry, temporal summation, three-item questionnaire. Can J Anaesth. 2021;68(12):1802-10.
5. Carvalho B, Cohen SE, Lipman SS, Fuller A, Mathusamy AD, Macario A. Patient preferences for anesthesia outcomes associated with cesarean delivery. Anesth Analg. 2005;101(4):1182-7.
6. Mkontwana N, Novikova N. Oral analgesia for relieving post-caesarean pain. Cochrane Database Syst Rev. 2015;2015(3):Cd010450.
7. Shanthanna H, Ladha KS, Kehlet H, Joshi GP. Perioperative Opioid Administration. Anesthesiology. 2021;134(4):645-59.
8. El-Boghdadly K, Desai N, Halpern S, Blake L, Odor PM, Bampoe S, et al. Quadratus lumborum block vs. transversus abdominis plane block for caesarean delivery: a systematic review and network meta-analysis(). Anaesthesia. 2021;76(3):393-403.
9. Shanthanna H, Weinberg G. Intravenous lidocaine, regional blockade, or both: considerations for multiple interventions involving local anaesthetics. Br J Anaesth. 2021;127(4):497-501.
10. Schug SA, Lavand'homme P, Barke A, Korwisi B, Rief W, Treede RD. The IASP classification of chronic pain for ICD-11: chronic postsurgical or posttraumatic pain. Pain. 2019;160(1):45-52.
11. Jin J, Peng L, Chen Q, Zhang D, Ren L, Qin P, et al. Prevalence and risk factors for chronic pain following cesarean section: a prospective study. BMC Anesthesiol. 2016;16(1):99.
12. Borges NC, de Deus JM, Guimarães RA, Conde DM, Bachion MM, de Moura LA, et al. The incidence of chronic pain following Cesarean section and associated risk factors: A cohort of women followed up for three months. PLoS One. 2020;15(9):e0238634.
13. Yimer H, Woldie H. Incidence and Associated Factors of Chronic Pain After Caesarean Section: A Systematic Review. J Obstet Gynaecol Can. 2019;41(6):840-54.
14. Wisner KL, Parry BL, Piontek CM. Clinical practice. Postpartum depression. N Engl J Med. 2002;347(3):194-9.
15. Eckerdal P, Georgakis MK, Kollia N, Wikström AK, Högberg U, Skalkidou A. Delineating the association between mode of delivery and postpartum depression symptoms: a longitudinal study. Acta Obstet Gynecol Scand. 2018;97(3):301-11.
16. Cox JL, Holden JM, Sagovsky R. Detection of postnatal depression. Development of the 10-item Edinburgh Postnatal Depression Scale. Br J Psychiatry. 1987;150:782-6.

17. Anders JJ, Lanzafame RJ, Arany PR. Low-level light/laser therapy versus photobiomodulation therapy. Photomed Laser Surg. 2015;33(4):183-4.
18. Clijsen R, Brunner A, Barbero M, Clarys P, Taeymans J. Effects of low-level laser therapy on pain in patients with musculoskeletal disorders: a systematic review and meta-analysis. Eur J Phys Rehabil Med. 2017;53(4):603-10.
19. Cheng K, Martin LF, Slepian MJ, Patwardhan AM, Ibrahim MM. Mechanisms and Pathways of Pain Photobiomodulation: A Narrative Review. J Pain. 2021;22(7):763-77.
20. JJ. A 2016;Pages. Accessed at American Society for Laser Medicine and Surgery at https://www.aslms.org/for-the-public/treatments-using-lasers-and-energy-based-devices/photobiomodulation.
21. Dutra LRDV, Araújo AMPdH, Micussi MT. Non-pharmacological therapies for postpartum analgesia: a systematic review. Brazilian Journal Of Pain. 2019.
22. Zimpel SA, Torloni MR, Porfírio GJ, Flumignan RL, da Silva EM. Complementary and alternative therapies for post-caesarean pain. Cochrane Database Syst Rev. 2020;9(9):Cd011216.
23. Khooshideh M, Latifi Rostami SS, Sheikh M, Ghorbani Yekta B, Shahriari A. Pulsed Electromagnetic Fields for Postsurgical Pain Management in Women Undergoing Cesarean Section: A Randomized, Double-Blind, Placebo-controlled Trial. Clin J Pain. 2017;33(2):142-7.
24. de Holanda Araujo AMP, de Sena KRR, da Silva Filho EM, Pegado R, Micussi M. Low-level laser therapy improves pain in postcesarean section: a randomized clinical trial. Lasers Med Sci. 2020;35(5):1095-102.
25. Mokmeli S, Khazemikho N, Niromanesh S, Vatankhah Z. The application of low-level laser therapy after cesarean section does not compromise blood prolactin levels and lactation status. Photomed Laser Surg. 2009;27(3):509-12.
26. Cooper SA, Desjardins PJ, Turk DC, Dworkin RH, Katz NP, Kehlet H, et al. Research design considerations for single-dose analgesic clinical trials in acute pain: IMMPACT recommendations. Pain. 2016;157(2):288-301.
27. Wengritzky R, Mettho T, Myles PS, Burke J, Kakos A. Development and validation of a postoperative nausea and vomiting intensity scale. Br J Anaesth. 2010;104(2):158-66.
28. Nisbet AT, Mooney-Cotter F. Comparison of selected sedation scales for reporting opioid-induced sedation assessment. Pain Manag Nurs. 2009;10(3):154-64.
29. Alvarenga MB, Francisco AA, de Oliveira SM, da Silva FM, Shimoda GT, Damiani LP. Episiotomy healing assessment: Redness, Oedema, Ecchymosis, Discharge, Approximation (REEDA) scale reliability. Rev Lat Am Enfermagem. 2015;23(1):162-8.
30. Childs C, Sandy-Hodgetts K, Broad C, Cooper R, Manresa M, Verdú-Soriano J. Risk, Prevention and Management of Complications After Vaginal and Caesarean Section Birth. J Wound Care. 2020;29(Sup11a):S1-s48.
31. Jahdi F, Khabbaz AH, Kashian M, Taghizadeh M, Haghani H. The impact of calendula ointment on cesarean wound healing: A randomized controlled clinical trial. J Family Med Prim Care. 2018;7(5):893-7.
32. Lu K, Luo X, Chen PY. Sample size estimation for repeated measures analysis in randomized clinical trials with missing data. Int J Biostat. 2008;4(1):Article 9.

APPENDIX 1: **Standard Operative Procedure for the Application of Bioflex LED Device and Laser probes**

As part of the proposed clinical trial, patients who have had C-section (CS) under spinal anesthesia will be randomized as Study or Control groups. The treatment sessions will be performed using the following equipment.

In all patients, a maximum of 5 PBMT sessions will be administered, either as ‘true’ or ‘sham’.

The frequency of these sessions will be: 4-6 hrs after the CS, morning (8 am), and evening (7 pm) of postoperative days (POD) 1 and 2. For all patients and for all treatment sessions, standardized procedures described below will be followed.

1. The sessions will be conducted by a trained research nurse at both sites.

2. Nurses will confirm patient ID and ongoing study consent. The nurse will be aware of the study group as per randomization (as they are unblinded) but will not reveal this to the patient.

3. The nurses will ensure the treatment session is conducted at a comfortable place and without disrupting any necessary clinical care.

4. All study patients will have a transparent (clear), adsorbent, waterproof dressing such as the following.

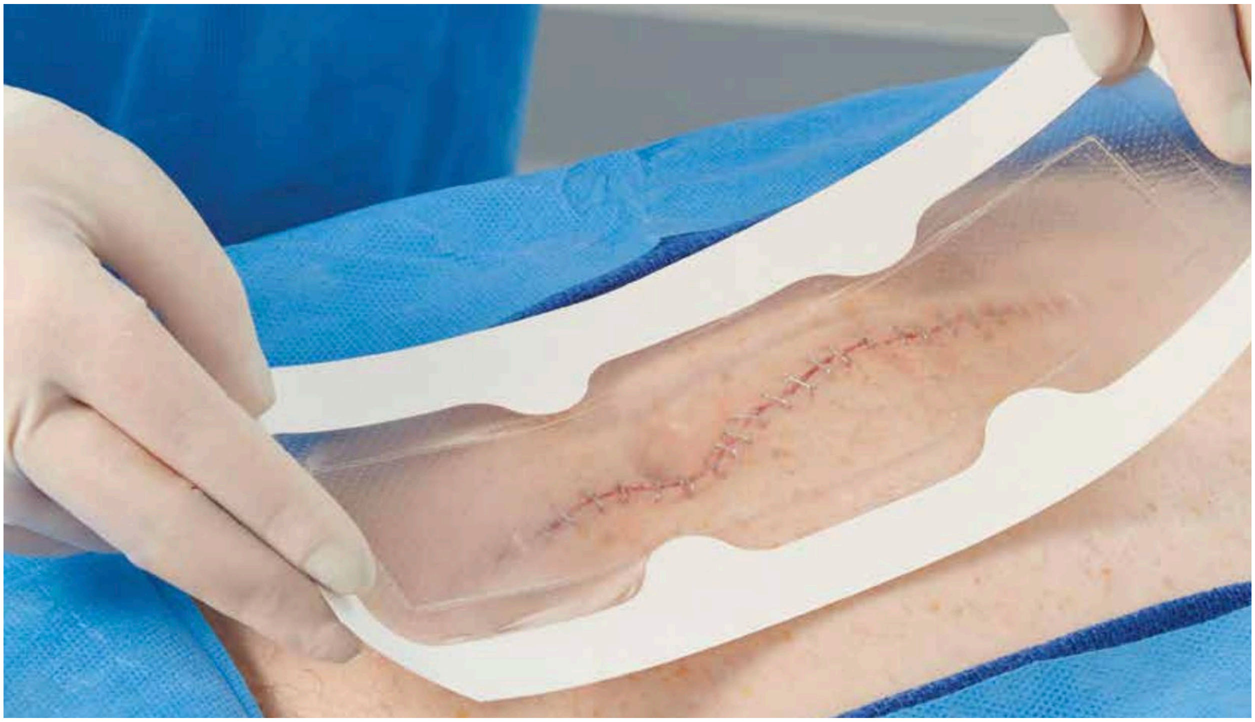

5. Before each session, the nurse will thoroughly wash her hands with soap and use a pair of sterile gloves (to check for Latex sensitivity or allergy).

6. The nurse will visually inspect the wound for any excessive discharge or bleeding or other abnormal signs and if present will notify the treating nurse or team.

7. Treatment session with LED device (DUO 240): the surface of the device will be cleaned using a germicidal disinfectant wipe such as Cavicide. The LED device will be applied in parallel and on the surgical incision slowly. In the study group patients' treatments will be applied for 7 minutes involving (red at 660 nm and near infrared at 840 nm). In the control group patients, non-effective dose of LED will be applied.

8. Treatment with LASER probes: This will be done soon after the LED therapy and will involve no direct contact of the probe. Before administering LASER therapy patient and the research nurse will wear protective eyeglasses cleaned before use. In the study group patients' spot treatment using the BIOFLEX LDR-100 laser probe (660 nm red light) and the LD1-200 laser probe (825 nm near infrared light), applied at the incision wound edges, for a total treatment time of 10 minutes. In the control group non-effective doses of LASER therapy will be applied.

9. After each session, the nurse will ask for any post-treatment patient concern and document the completion of each treatment session as complete, incomplete, or did not happen.

APPENDIX 2: **REEDA SCALE FOR ASSESSMENT OF WOUND HEALING**

Adapted from Childs C, Sandy-Hodgetts K, Broad C, Cooper R, Manresa M, Verdú-Soriano J. Risk, Prevention and Management of Complications After Vaginal and Caesarean Section Birth. J Wound Care. 2020 Nov 1;29(Sup11a):S1-S48.

	**Redness**	**Edema**	**Ecchymosis (discoloration of skin due to underlying blood)**	**Discharge**	**Approximation**
**0**	None	None	None	None	Closed
**1**	Mild: less than 0.5cm from each side of the wound edge	Mild: Less than 1cm from each side of the wound edge	Mild: Less than 1cm from each side of the wound edge	Serous	Skin separation 3 mm or less

**2**	Moderate: 0.5cm to 1cm from each side of the wound edge	Moderate: 1cm to 2cm from each side of the wound edges	Moderate: 1cm to 2cm from each side of the wound edges	Serosanguinous	Skin and subcutaneous fat separated
**3**	Severe: More than 1cm from each side of the wound edges	Severe: More than 2cm from each side of the wound edges	Severe: More than 2cm from each side of the wound edges	Bloody and purulent	Skin, subcutaneous fat and fascia separated

INFORMATION/CONSENT FORM (PARTICIPANT)

**Title of the Study:**	Application of photo-biomodulation as part of multimodal analgesia to improve pain relief and wound healing for patients having elective Caesarean section: a randomized controlled trial
**Principal Investigator:**	Dr. Harsha Shanthanna, MD/SJHH
**Co-Investigators:**	Dr. Daniel Cordovani, MD/HHSC; Dr. Lea Luketic, MD/SJHH and Dr. Shapna Sharma, MD/HHSC
**Study Sponsor:**	Department of Anesthesia, McMaster University and Research Institute of St Joseph's Hospital

You are being invited to participate in a research study conducted by Dr. Harsha Shanthanna because you are scheduled for an elective C-section.

In order to decide whether or not you want to be a part of this research study, you should understand what is involved and the potential risks and benefits. This form gives detailed information about the research study, which will be discussed with you. Once you understand the study, you will be asked to sign this form if you wish to participate. Please take your time to make your decision. Feel free to discuss it with your friends and family, or your family physician.

WHY IS THIS RESEARCH BEING DONE?

C-section is the most common surgical procedure performed in Canada and is associated with moderate to severe pain after the surgery. If the pain is not adequately managed , it may make it difficult for you to be comfortable and participate in activities. Additionally, it may contribute to postpartum depression and persistent pain. Typically a combination of medications such as Tylenol, anti-inflammatories are routinely used after surgery, patients can still experience distressing pain, which can interfere with newborn bonding. Although generally safe and used as rescue medications for severe pain, opioids are preferentially avoided because of side effects and newborn safety.

In view of all these reasons, there is a greater need for non-pharmacological approaches for pain relief. One non-pharmacological approach is a light therapy that uses a low-level laser combined with light-emitting diodes (photo-biomodulation therapy [PBMT]), which together work on pain and wound healing. PBMT has been used in many musculoskeletal conditions and in some surgeries, but has not been well-studied in pain after C-section. This study is being done to examine the effects of PBMT on pain and healing after elective C-section.

WHAT IS THE PURPOSE OF THIS STUDY?

The primary purpose of this study is to evaluate the effect of PBMT, on post-surgical pain after elective C-section. The researchers will also collect data on opioid use, opioid-related side effects, wound healing, persistent pain, post-partum depression, and any adverse events related to PBMT.

WHAT WILL MY RESPONSIBILITIES BE IF I TAKE PART IN THE STUDY?

If you volunteer to participate in this study, we will ask you to do the following things:

While in hospital:

You will be randomly assigned (like a flip of a coin) to have either active PBMT, or inactive PBMT (placebo group). Only the research nurse will know which treatment you are getting. Patients, doctors, hospital nurses, and other research staff will not know. After your surgery, the wound will be covered with a clear plastic bandage called Tegaderm. This product stays in place and prevents any contact with any clothing, hands, or any equipment, including the ones used in the study. The trained research nurse will administer PBMT sessions, as one session on the evening of surgery, one session in the morning and evening each for the next two days after surgery. Each session will involve LED therapy (7 minutes) applied parallel to the abdominal incision scar, followed by spot treatment using the BIOFLEX LDR-100 laser probe and the LD1-200 laser probe applied at the incision wound edges, for a total treatment time of 10 minutes. Placebo lasers will have non-effective levels of light and laser. You will have access to all usual pain medications and hospital care regardless of your treatment group.

Measurements in hospital:
A research person will come to visit and collect pain measurements at the following time points.
Day of surgery: Once in the evening and ask you to rate your pain.
Postoperative day 1: Once in the morning, noon, and evening to ask you to rate your pain.
Postoperative day 2: Once in the morning, noon, and evening to ask you to rate your pain.

After you leave hospital:
At 6 weeks: A research assistant will meet with you at the time of your 6-week check up to do the following:

1. Assess your wound to check for healing
2. Ask you if had any adverse effects associated with PBMT
3. Ask you if you have persistent pain (yes/no question)
4. Ask you to fill out a questionnaire to assess for postpartum depression

At 3 months: A research assistant will meet with you by telephone

1. Ask you about presence of chronic pain
2. Ask you about any wound healing problems, or wound infection
3. Ask you to fill out a questionnaire to assess for postpartum depression

WHAT ARE THE POSSIBLE RISKS AND DISCOMFORTS?
PBMT has no particular major risks identified in literature. There is potential for minor pain or discomfort, skin irritation or redness, lightening or darkening of skin or tattoos, freckle loss or lightening of moles, and skin pigment changes. During the use of LASER probes, protective eyeglasses will be worn to avoid any potential injury to the eye.

HOW MANY PEOPLE WILL BE IN THIS STUDY?
There will be 180 patients from two sites, which include St Joseph's Hospital and McMaster University Medical Centre.

WHAT ARE THE POSSIBLE BENEFITS FOR ME AND/OR FOR SOCIETY?
We cannot promise any personal benefits to you from your participation in this study. However, possible benefits include less pain and improved wound healing. Your participation may help other people undergoing elective C-section with pain and wound healing in the future.

IF I DO NOT WANT TO TAKE PART IN THE STUDY, ARE THERE OTHER CHOICES?
It is important for you to know that you can choose not to take part in the study. Choosing not to participate in this study will in no way affect your care or treatment.

WHAT INFORMATION WILL BE KEPT PRIVATE?
Your identifiable data will not be shared with anyone except with your consent or as required by law. All personal information such as your name, address, phone number, OHIP number, family physician's name will be removed from the data and will be replaced with a number. A list linking the number with your name will be kept in a secure place, separate from your file. The data, with identifying information removed, will be securely stored in a password protected computer in a locked research office. The information for this research will be kept for 10 years and then destroyed.

It is possible that representatives of the Hamilton Integrated Research Ethics Board, this institution and affiliated sites may consult your original (identifiable) research data and medical records to check that the information collected for the study is correct and follows proper laws and guidelines.

If the results of the study are published, your name will not be used and no information that tells us your identity will be released or published without your specific permission to reveal your information. However, it is important to note that this original signed consent form and the data which follows may be included in your health record.

Your participation in this study will be recorded in your electronic health record (EHR), also called a medical record, at Hamilton Health Sciences. If you participate, some of the information about you that is collected for this study, including the results of tests described in this consent form, will be stored in your EHR and accessible to others working at this hospital (like your current and future health care provider(s)). This hospital may share patient information stored in its EHR with other hospitals and healthcare providers in Ontario. In addition, any person or company to whom you give access to your medical record may have access to this information. The study team can tell you what information about you will be stored electronically, and what may be shared outside of this hospital.

CAN PARTICIPATION IN THE STUDY END EARLY?
If you volunteer to be in this study, you may stop the study at any time. This will in no way affect the quality of care you receive at this institution. You have a choice to remove your data from this study. You may refuse to answer any questions you don't want to answer and still stay in the study.

You will be informed of any new information that might change your decision to continue in this study. If you do change your mind and wish to withdraw, let anyone on the research study team know and you will be withdrawn immediately. You can also contact Toni Tidy, Research Coordinator, at (905) 525-9140 ext. 21737 or tonitidy@mcmaster.ca

WILL I BE PAID TO PARTICIPATE IN THIS STUDY?
You will not be paid to participate in this study.

WILL THERE BE ANY COSTS?
Your participation in this research project will not involve any additional costs to you or your health care insurer.

WHAT HAPPENS IF I HAVE A RESEARCH-RELATED INJURY?

If you suffer an injury from participation in this study, medical care will be made available to you by your study doctor, or you will be referred for appropriate medical care.

IF I HAVE ANY QUESTIONS OR PROBLEMS, WHOM CAN I CALL?

Dr. Daniel Cordovani can be contacted at McMaster University Medical Centre at (905) 521-2100 ext. 75154.
Dr. Harsha Shanthanna can be contacted at St. Joseph's Healthcare Hamilton at (905) 522-1155 ext. 33853.
or Toni Tidy can be contacted at McMaster University at 905-525-9140 ext. 21737

CONSENT

<u>Participant:</u>
I have read the preceding information thoroughly. I have had an opportunity to ask questions, and all my questions have been answered to my satisfaction. I agree to participate in this study. I understand that I will receive a signed copy of this form. By signing this form, I do not give up any of my legal rights.

Name	Signature	Date

<u>Person Obtaining Consent:</u>
I have discussed this study in detail with the participant. I believe the participant understands what is involved in this study.

Name	Signature	Date

<u>Witness:</u>
I was present when the information in this form was explained and discussed with the participant. I believe the participant understands what is involved in this study.

Name	Signature	Date

This study has been reviewed by the Hamilton Integrated Research Ethics Board (HIREB). The REB is responsible for ensuring that participants are informed of the risks associated with the research, and that participants are free to decide if participation is right for them. If you have any questions about your rights as a research participant, please call The Office of the Chair, at 905-521-2100 extension 42013.
